# Corrosion Resistance of Electrochemically Synthesized Modified Zaccagnaite LDH-Type Films on Steel Substrates

**DOI:** 10.3390/ma14237389

**Published:** 2021-12-02

**Authors:** Michael Kahl, Teresa D. Golden

**Affiliations:** Department of Chemistry, University of North Texas, Denton, TX 76203, USA; mkahl87@gmail.com

**Keywords:** zaccagnaite, LDH, corrosion protection, ceramic matrix composites, electrochemical deposition

## Abstract

Modified zaccagnaite layered double hydroxide (LDH) type films were synthesized on steel substrates by pulsed electrochemical deposition from aqueous solutions. The resulting films were characterized by X-ray diffraction, scanning electron microscopy/X-ray dispersive spectroscopy, and Fourier transform infrared spectroscopy. Structural characterization indicated a pure layered double hydroxide phase; however, elemental analysis revealed that the surface of the films contained Zn:Al ratios outside the typical ranges of layered double hydroxides. Layer thickness for the deposited films ranged from approximately 0.4 to 3.0 μm. The corrosion resistance of the film was determined using potentiodynamic polarization experiments in 3.5 wt.% NaCl solution. The corrosion current density for the coatings was reduced by 82% and the corrosion potential was shifted 126 mV more positive when 5 layers of modified LDH coatings were deposited onto the steel substrates. A mechanism was proposed for the corroding reactions at the coating.

## 1. Introduction

Steels are utilized in many applications for a variety of industries, including architectural/civil engineering, medical equipment, oil and gas, food and drink processing/storage, water treatment/transport, automotive, and pharmaceutical [1,2,3,4,5,6,7]. Stainless steel has good corrosion resistance in various corrosive environments, with resistance derived from its chromium component. A minimum of 12% chromium allows for the formation of a protective chromium oxide layer. The oxide layer is self-repairing in oxygen-rich environments [8]. However, stainless steels are susceptible to localized corrosion in various environments [9,10]. Stainless steels are often coated to prolong lifetime when utilized in a chloride environment [11,12]. Many types of coatings have been developed and recently there has been a shift towards more environmentally friendly coatings. There have been several studies showing the potential of using layered double hydroxides (LDHs) which are anionic clays as coatings, for metal or alloy substrates [13,14,15,16].

LDHs are a class of layered anionic clays derived from the natural clay hydrotalcite. They are comprised of metal hydroxide layers with anions and water in the interlayer regions between the metal sheets. LDH is represented by the formula [M^2+^_1−x_M^3+^_x_(OH)_2_][A^n−^]_x/n_ zH_2_O, where M^2+^ and M^3+^ are divalent and trivalent metal cations, respectively. A^n−^ is an anion such as CO_3_^2−^ or NO_3_^−^, x is the M^3+^/(M^2+^ + M^3+^) ratio, and z is the number of associated water molecules [17]. The positive charge is derived from the substitution of divalent ions with trivalent ions in brucite-like metal hydroxide. This positive charge is balanced by interlayer anions which can be exchanged. A divalent—to-trivalent ratio (M^2+^:M^3+^) between 4:1 and 2:1 is considered the range for a material to be reliably classified as an LDH, although there are exceptions [17,18,19].

LDHs are thermally stable, inexpensive and eco-friendly and are known for their high anion exchange capacities and adsorption properties. Because of their anion exchange properties, LDHs can be used in corrosion resistant coatings. The coating can undergo anion exchange to trap chloride ions and prevent them from attacking the substrate [20]. Tedim et al. showed that an LDH-NO_3_ coating can lower the permeability of chlorides to the substrate, delaying coating degradation and improving corrosion resistance. The LDH coating acted as a ‘nanotrap’ for chloride ions [20]. LDH coatings also exhibit barrier properties, especially when the thickness is in the micron range [21]. LDH films can be spin-coated to produce thicker films improving corrosion protection by blocking penetration of the chloride ions [22]. Hydrotalcite films have also been prepared to provide barrier (passive) protection against chloride attack due to a decrease in pinhole type defects [23]. The growth mechanism along with the corrosion mechanism for the Mg-Al hydrotalcite films have been studied on magnesium alloys [24,25,26]. Mg-Al-CO_3_ LDH films were synthesized by a combined co-precipitation and hydrothermal process on AZ31 alloy [27]. These films increased the corrosion protection of the substrate but required a 48 h synthesis, 12 h aging process and a 24–48 h heat treatment in an autoclave. Films containing both crystalline Mg(OH)_2_ and Mg-Al-CO_3_ LDH were generated by a steam coating method on magnesium alloy AZ31 at temperatures up to 453 K [28]. These films displayed excellent corrosion resistance in 5 wt.% NaCl solution. Films grown by in situ crystallization have exhibited self-healing properties in 3.5 wt.% NaCl [29].

Electrochemical deposition is another technique for the in situ generation of films on various substrates [30]. Electrodeposition of thin films is an attractive technique because of the low cost, simple setup, short synthesis time, and ability to deposit on large or unconventional substrate shapes [11]. Furthermore, there is greater control over film properties and deposition rate by changing the deposition parameters [31,32,33]. Zaccagnaite is a hexagonal Zn-Al-LDH, a substituted variant of hydrotalcite, and represented by the formula Zn_4_Al_2_(OH)_12_[CO_3_] 3H_2_O [19]. It has a metal ratio of 2:1 in natural mineral formations and various ratios have been synthetically prepared. There has been very little work done on the direct electrodeposition of LDH-type coatings for corrosion protection. Yarger et al. electrodeposited Zn-Al-NO_3_ films onto gold-coated glass substrates with a nitrate solution containing Zn^2+^ and Al^3+^ ions [34]. An optically transparent Li-Al-CO_3_ LDH was electrochemically deposited onto AZ31 substrate from a Li^+^/Al^3+^ aqueous solution [21]. The coating provided excellent corrosion protection to the substrate but synthesis of the electrolyte solution required many steps. Wu et al. deposited Zn-Al-NO_3_ films onto AZ91D Mg alloy substrate in a Zn^2+^/Al^3+^ aqueous solution [35]. The LDH coating showed great corrosion resistance and improved adhesion to the substrate. For LDH electrodeposition, the electrochemical generation of OH^−^ species via a reduction reaction of the nitrate ions is important since the increase in local pH facilitates the formation of the LDH films at the substrate surface [34]. However, for Zn-Al LDH deposition, other Zn phases can interfere with the LDH deposition if the deposition conditions are not precisely controlled. If the deposition potential is too negative, Zn and Zn hydroxide species may form and if the deposition potential is too positive Zn oxide species may deposit [36]. Additionally, if the OH^−^ production rate slows, Al(OH)_3_ precipitate can compete with LDH deposition. If the OH^−^ production rate is kept high enough, then LDH directly forms [37]. Since the M(II) and M(III) ions are rapidly consumed near the electrode surface, we therefore propose a pulse deposition method to produce the LDH type coatings. Pulsing the deposition conditions resets the deposition process allowing regeneration of the OH^−^ species with each pulse step and time for diffusion of the metal species. This could improve protection capacity of the resulting LDH-type coatings. In this study, a facile pulsing method was developed to electrochemically deposit modified zaccagnaite films onto a steel substrate at room temperature. These films are modified zaccagnaite materials for two reasons. First, the elemental ratio of Zn:Al is outside the typical range for LDH and secondly the carbonate group has been exchanged with nitrate. A designed step potential method was used to synthesize the films. A short pulse deposition duration followed by drying of the film was repeated up to five times to mitigate the fracturing of the film which commonly occurs in hydroxide and oxide films prepared from aqueous solutions. These films were characterized and tested for their corrosion resistance in 3.5 wt.% NaCl aqueous solution.

## 2. Materials and Methods

### 2.1. Film Synthesis

The substrates were stainless-steel (430) discs from Ted Pella, Inc (Redding, CA, USA). with a diameter of 10 mm, a thickness of 0.76 mm and an area of 1.77 cm^2^. The substrates contained Fe, <0.12% C, 16–18% Cr, <0.75% Ni, <1.0% Mn, <1.0% Si, <0.040% P, and <0.030% S by weight. The discs were ground with SiC 320 grit paper (LECO, St. Joseph, MI, USA) and then degreased by sonication in acetone, and then attached to coiled copper wire leads with conductive silver epoxy (EPO-TEK, Billerica, MA, USA). Once dry, the substrate was mounted in epoxy utilizing molds. After 24 h curing, the mounted electrodes were ground successively with 320, 400, and 600 grits of SiC paper then polished with 1 μm diamond suspension on a felt polishing pad followed by ultrasonication in ethanol. The resulting substrate surface had a mirror finish with an embedded uniform, flat, even surface with the epoxy.

The electrolytic solution was prepared by dissolving a 2:1 molar ratio of Zn^2+^ to Al^3+^ ions in DI water. Aluminum nitrate nonahydrate (Al(NO_3_)_3_•9H_2_O, Alfa Aesar, Tewksbury, MA, USA) was the aluminum source, zinc nitrate hexahydrate (Zn(NO_3_)_2_•6H_2_O, Alfa Aesar, Tewksbury, MA, USA) was the source of zinc, and potassium nitrate (KNO_3_, Fisher Scientific, Walham, MA, USA) was used as the electrolyte to help facilitate the formation of modified zaccagnaite film at the electrode surface. The electrolytic concentration of each compound was 0.02 M Zn(NO_3_)_2_**•**6H_2_O, 0.01 M Al(NO_3_)_3_•9H_2_O, and 0.2 M KNO_3_. The final pH of the deposition solution was 3.8. An EG&G Princeton Applied Research (PAR) Model 273A potentiostat/galvanostat (Oak Ridge, TN, USA) was used to electrochemically deposit films. The depositions were performed at room temperature utilizing a three-electrode configuration. Due to the fact that carbonate ions (carbon dioxide dissolution) have an exceptionally high affinity to the LDHs [18], the deposition solution was purged with nitrogen for 20 min prior to film fabrication in order to minimize carbonate contamination and allow nitrate insertion in the interlayers. The working electrode was the polished stainless-steel disc, a platinum mesh was used as the counter electrode and the reference electrode was a saturated calomel electrode (SCE) (Fisher Scientific, Walham, MA, USA). Films were deposited in multiple steps. A step potential method was used for film deposition. The applied potential started at −1.5 V vs. SCE for 5 sec and then stepped to −1.0 V vs. SCE for 20 sec; this cycling was done for a total of 50 sec and represented deposition of one layer. Each layer was electrodeposited and allowed to dry undisturbed before the next layer was added. A total of 1, 2, and 5 layers were deposited, (1L, 2L, 5L).

### 2.2. Characterization

The structure and phase composition of the modified zaccagnaite films were identified by X-ray diffraction (XRD) using a Siemens D500 diffractometer (KSA, Aubrey, TX, USA) with Cu Kα radiation (λ = 1.5405 Å) as the source in a standard Bragg-Brentano configuration. The x-ray tube was operated at 35 kV and 24 mA. Each sample was scanned from 2 to 40° (2θ), at a step size of 0.05° and a dwell time of 1.0 s. The surface morphology of the films was characterized by scanning electron microscopy (SEM) with an X-ray dispersive spectroscopy (EDX) attachment (FEI Quanta 200 ESEM, Hillsboro, OR, USA). A spot size of 3.0 and an accelerating voltage of 25 kV were used for SEM. Film thickness measurements were performed with a Veeco Dektak 150 stylus profilometer (Plainview, NY, USA). A Perkin Elmer Spectrum One FT-IR Spectrophotometer (Waltham, MA, USA) was used to analyze the composition of the films. The films were scraped off with a blade and ground up further before being placed onto an ATR stage. Each sample was scanned 16 times at a wavenumber range of 4000–450 cm^−1^.

### 2.3. Immersion Test and Corrosion Measurements

Potentiodynamic polarization measurements and immersion tests were performed in 3.5 wt.% NaCl aqueous solutions at room temperature. Electrochemical measurements were conducted with an EG&G Princeton Applied Research (PAR) Model 273A potentiostat/galvanostat. The coated film and a SCE electrode were used as the working and reference electrodes, respectively. Two graphite rods were used as the counter electrodes for polarization measurements. Each sample was immersed in the NaCl solution for 30 min before polarization curves were measured with respect to open circuit potential (OCP) at a scan rate of 1 mV/s. The polarization resistance, R*_p_*, was the slope value obtained from linear polarization (LPR) scanning ±150 mV with respect to *E_corr_*. *E_corr_* was determined as the point of intersection of the anodic and cathodic polarization branches. To obtain a more accurate estimation of i*_corr_*, the cathodic polarization region was used since the anodic region contained current density oscillations. In the tafel plot, a horizontal line was drawn at the E*_corr_* value and another horizontal line was drawn 100 mV cathodic from E*_corr_*. A slope line was drawn from the 100 mV meeting point on the cathodic branch to intersect with the E*_corr_* line. The point of intersection was taken as the value of i*_corr_* [38,39,40]. Immersion tests were performed in 3.5 wt.% NaCl solution at room temperature for up to 168 h to examine the long-term corrosion resistance of the zaccagnaite-coated films.

## 3. Results and Discussion

### 3.1. Film Deposition Mechanism

The electrochemical synthesis of hydroxides can occur by electrogeneration of base via a nitrate reduction at the working electrode interface (Equation (1)) [41].

NO_3_^−^ + H_2_O + 2e^−^ → NO_2_^−^ + 2OH^−^ (1)

The deposition solution contains nitrates from the supporting electrolyte (0.2 M KNO_3_) and also as the salt of the divalent/trivalent cations. By applying a cathodic potential to the electrode, the pH at the electrode surface increases because of the consumption of H^+^ and generation of OH^−^. The higher the applied cathodic potential, the faster the pH increases; this increase in local pH leads to metal hydroxide precipitation at the electrode surface. Precipitation of metal hydroxides at the electrode consumes OH^−^ and lowers the pH (Equation (2)) [36,37,42].

Zn^2+^ + Al^3+^ + *n*OH^−^ + NO_3_^−^ → Zn-Al LDH (2)

Characterization of the surface helps identify the structural and chemical properties of the electrodeposited coating.

### 3.2. Structural Characterization

**Figure 1:**
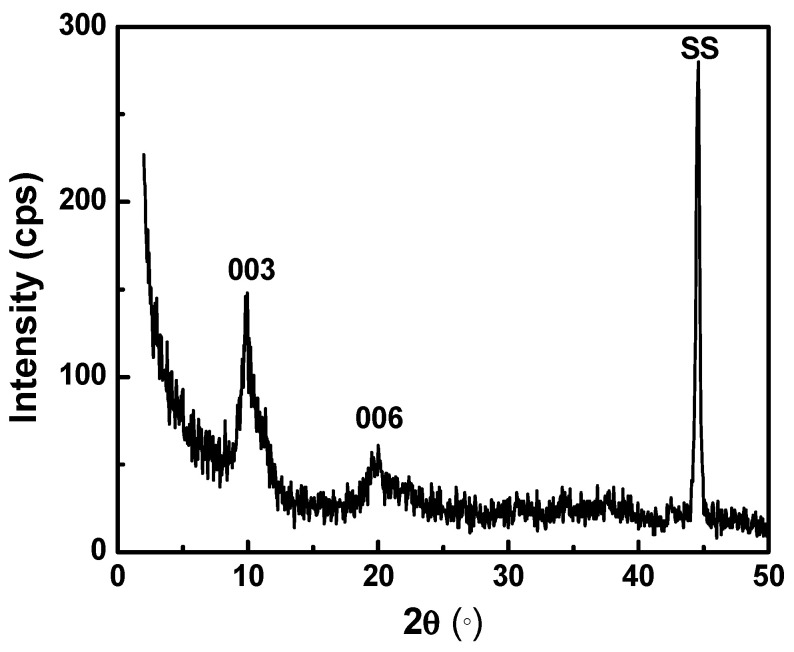
X-ray diffraction pattern of an electrodeposited modified zaccagnaite coating on stainless steel (SS) substrate.

The x-ray diffraction pattern of the modified zaccagnaite film deposited on the substrate is displayed in Figure 1. The film was grown during one continuous deposition in order to obtain enough material for characterization. The peaks at 9.89° and 20.00° (2θ) represent the characteristic (003) and (006) reflections for LDH. The peak at 44.61° corresponds to the substrate. A basal spacing of 0.89 nm was calculated from the most intense peak at 9.89° according to Bragg’s equation. This value is in agreement with previous studies [34,35,42]. The absence of non-basal reflections is evidence that the film is composed of highly oriented platelets [22,43]. The peaks may be of low intensity due to the thickness of the films (0.43 to 2.8 μm) and transparency of the lighter weight elements to X-rays, as well as some slight amorphous nature. Furthermore, the diffraction peaks are slightly broad due to poor crystallinity as also seen with previous electrosynthesized LDH films [37].

**Figure 2:**
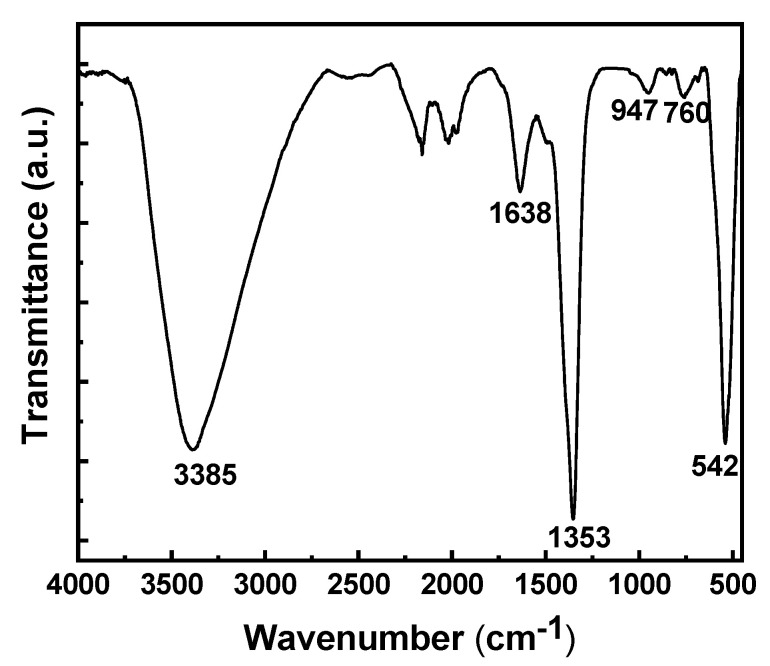
The FTIR spectrum of an electrodeposited modified zaccagnaite film.

The FT-IR spectrum for the deposited film is displayed in Figure 2. The spectrum confirms the presence of hydroxides with only water and nitrate ions in the interlayer region. The broad peak at 3385 cm^−1^ corresponds to O-H stretching of hydroxide and water in the interlayer region. The peak at 1638 cm^−1^ shows the bending vibration of the interlayer water molecules. The peak at 1353 cm^−1^ represents the asymmetric stretching of nitrate ions in the interlayer region [34]. The peaks at 947, 760 and 542 cm^−1^ are associated with Al-O and Zn-O stretching modes [44,45,46,47]. The peaks from 2600 to 1800 cm^−1^ are from the diamond ATR surface [48].

**Figure 3:**
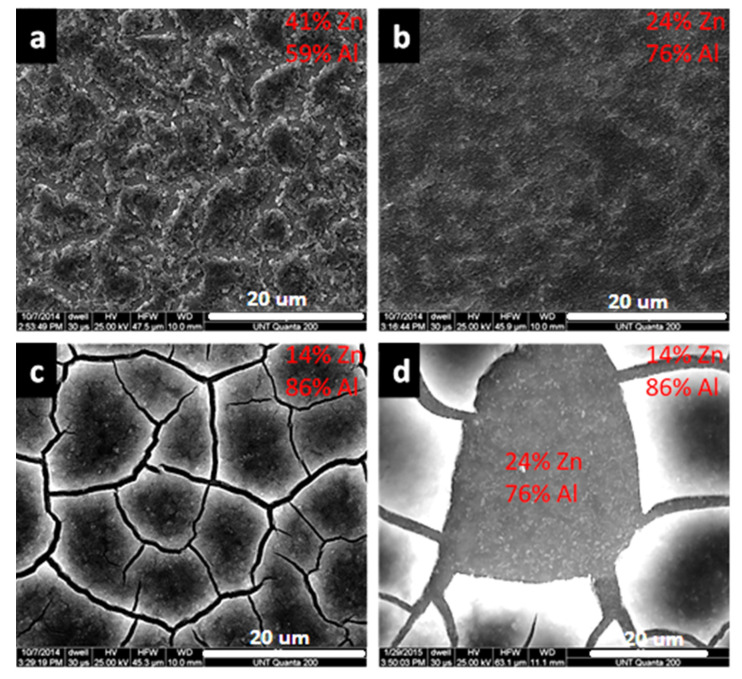
SEM images of modified zaccagnaite films for (**a**) 1L, (**b**) 2L, (**c**) 5L, and (**d**) 5L film with a portion of top phase removed (L = layer). (Image bars are 20 μm).

Figure 3 shows the surface morphology of modified zaccagnaite films from a one layer (1L), two layer (2L), and five layer (5L) successive depositions. The surface of the 1L film (Figure 3a) shows a flattened disorganized coating with uneven coverage. The elemental ratio of Zn:Al measured by EDX on the surface of the film is approximately 1:1.2. Table 1 lists the elemental ratios and film thicknesses for the coatings. The SEM image of the 2L film (Figure 3b) shows a fairly homogenous surface without the flaws observed in the 1L films (Figure 3a). The double deposition reduced the uneven coverage. The elemental ratio of Zn:Al on the surface of the 2L film is approximately 1:2.8. The 5L film (Figure 3c) exhibits a surface which is different from both the 1L and 2L films. The elemental ratio of the 5L film’s surface is approximately 1:5, while this film does not have the uneven coverage in material as seen in the 1L film, there are cracks observed. This cracking and shrinkage of the LDH coatings has been observed by other researchers and is typically attributed to dehydration of the coating in atmosphere and shown to be spontaneous upon drying (Equation (3)) [34,49,50,51]. Lu et al. showed that a dehydration/rehydration process was reversible by monitoring the LDH gallery spacings with XRD for Ni-Fe LDH coatings [52]. Gualandi et al. measured the gallery d-spacings of several LDH coatings with XRD and noted that Zn-Al LDH had the highest hydration levels (larger d-spacing) compared to Ni-Al LDH and Co-Al LDH coatings [37] and this cracking could even be observed in the SEM cross-sections [53]. Others have shown that hydrogen gas evolution during the deposition can occur at too high of cathodic potentials [35]. Even with the cracking process, adhesion was still good for the coatings [54].

[Zn^2+^_1−x_Al^3+^_x_(OH)_2_][NO_3_^−^]_x_ zH_2_O → [Zn^2+^_1−x_Al^3+^_x_(OH)_2_][NO_3_^−^]_x_ + zH_2_O (3)

In Figure 3d, it is apparent that these cracks do not penetrate all the way to the substrate. Two separate phases can be observed, a fractured phase upon a continuous underlying phase. The surface of the underlying phase in Figure 3d has slightly more zinc (1:4 Zn:Al ratio) than the 5L film. Previous research has shown that LDH with an acceptable divalent:trivalent cation ratio is only formed during a certain time frame, dependent on the deposition potential, formation pH of the divalent cation hydroxide and divalent:trivalent cation ratio. At longer synthesis times, an aluminum dominated hydroxide phase is formed [48]. These electrochemical deposited coatings show the same trend, with increasing Al composition corresponding to increasing deposition times.

Film thickness measurements for 1L, 2L, and 5L films are reported in Table 1. The film thickness does not increase proportionally with the number of deposition layers. The lack of linearity may be due to deposition defects caused by dehydration of previous layers. Furthermore, the rate of film growth may change due to the differences in initial growth on the substrate versus growth on the previously deposited film. The formation of two sequential phases may also cause irregular film growth rate. The conductivity variation in each layer and the non-conducting nature of the zinc and aluminum hydroxide film will also contribute to the nonlinear growth rate.

**Figure 4:**
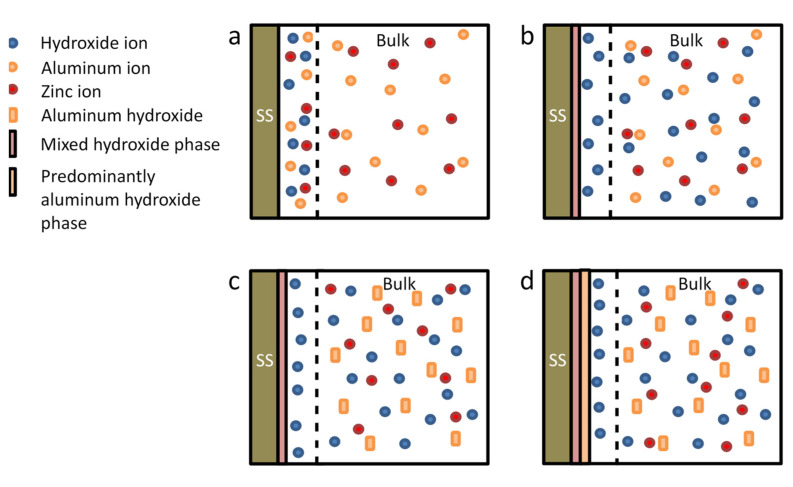
Film formation process of the mixed hydroxide phase and top aluminum hydroxide layer for the electrodeposited zaccagnaite coating on stainless-steel substrate, (**a**) generation of hydroxides at the electrode, (**b**) deposition of metal hydroxides and diffusion of unconsumed hydroxides away from the electrode surface, (**c**) pH increases in the bulk solution resulting in aluminum hydroxide precipitation, and (**d**) aluminum hydroxide precipitate formed in bulk deposits onto mixed hydroxide layer.

**Figure 5:**
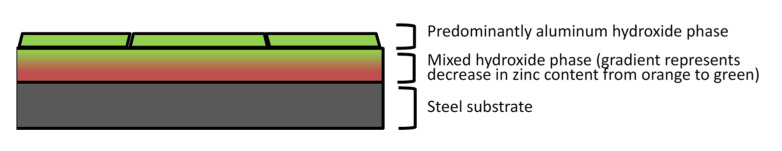
Changing film composition from the substrate to the outer top layer for the electrodeposited zaccagnaite coating.

Figure 4 shows a schematic representing the changing deposition mechanism where initially hydroxides are generated at the electrode surface. When the pH increases sufficiently at the electrode, metal hydroxides began to precipitate and any hydroxides not consumed move into the bulk of the solution. The diffusion of hydroxides away from the electrode increases the pH of the solution resulting in the precipitation of aluminum hydroxide. Aluminum hydroxide forms at approximately pH 4 and zinc aluminum LDH precipitates at approximately pH 6 [35,55]. The aluminum hydroxide precipitate coats the mixed hydroxide phase. This deposition process results in the film structure depicted in Figure 5, which is comprised of a mostly aluminum hydroxide phase on top of a mixed hydroxide phase. The zinc content of the mixed hydroxide phase is higher at the substrate interface and decreases as the film thickness increases. Zinc hydroxide is slightly more soluble (K_sp_ = 3 × 10^−17^) than aluminum hydroxide (K_sp_ = 3 × 10^−34^), also leading to a higher aluminum hydroxide solid on the surface.

### 3.3. Corrosion Resistance

**Figure 6:**
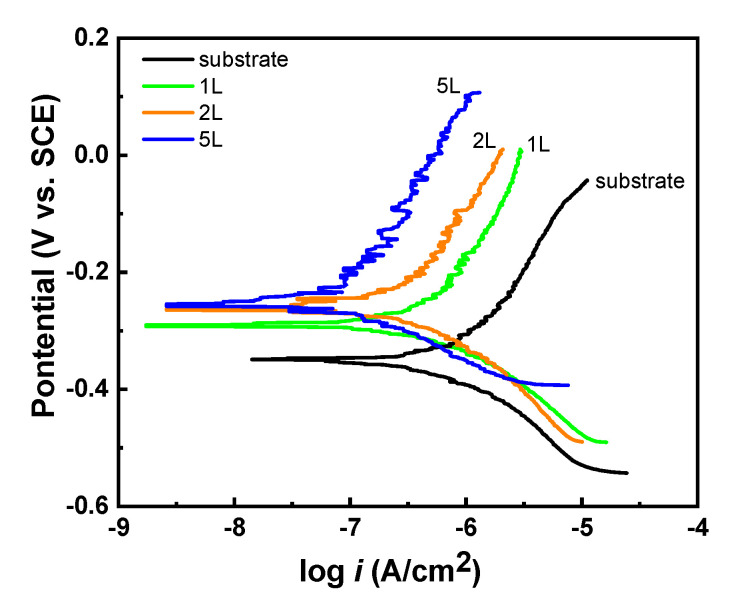
Potentiodynamic polarization curves of the substrate, and 1L, 2L, 5L modified zaccagnaite films measured in a 3.5 wt.% NaCl solution.

The potentiodynamic polarization curves of the bare substrate, 1L, 2L, and 5L as-deposited films measured in 3.5 wt.% NaCl solution are shown in Figure 6. The corrosion potential (*E_corr_*), corrosion current density (*i_corr_*), and polarization resistance (*R_p_*) data for each sample are listed in Table 2. Deposition of the modified zaccagnaite film resulted in a positive shift in the *E_corr_* and a decrease in the *i_corr_* compared to the bare substrate (better corrosion resistance). The 1L film had the greatest effect on *E_corr_*, shifting almost 100 mV in the positive direction while the 2L and 5L films had a continued shift in *E_corr_* by smaller increments of approximately 30 and 3 mV, respectively. The *i_corr_* decreased from 0.66 μA/cm^2^ to 0.61, 0.38, and 0.12 μA/cm^2^ for the 1L, 2L, and 5L films, respectively. Polarization resistance, *R_p_*, increased with the number of deposited layers resulting in the 5L film having a *R_p_* an order of magnitude larger than the substrate *R_p_*. The beginning of a pitting potential can be observed for the bare substrate sample at the end of the polarization curve around −50 mV. For each deposition layer, an increasing barrier property is observed in the anodic branch of potentiodynamic curves. These polarization measurements indicate that the film provides a barrier to the transport of aqueous species to the substrate so that the ability of the chloride ions to attack the substrate is reduced. This barrier increases and defects are minimized as more layers are deposited. Some current density oscillation is observed in the anodic branch of the polarization curves. The current density oscillation becomes greater as the number of deposited layers increases. This may be due to the dissolution of hydroxides from the coating surface or even some interlayer exchange of species between the film and the solution.

**Figure 7:**
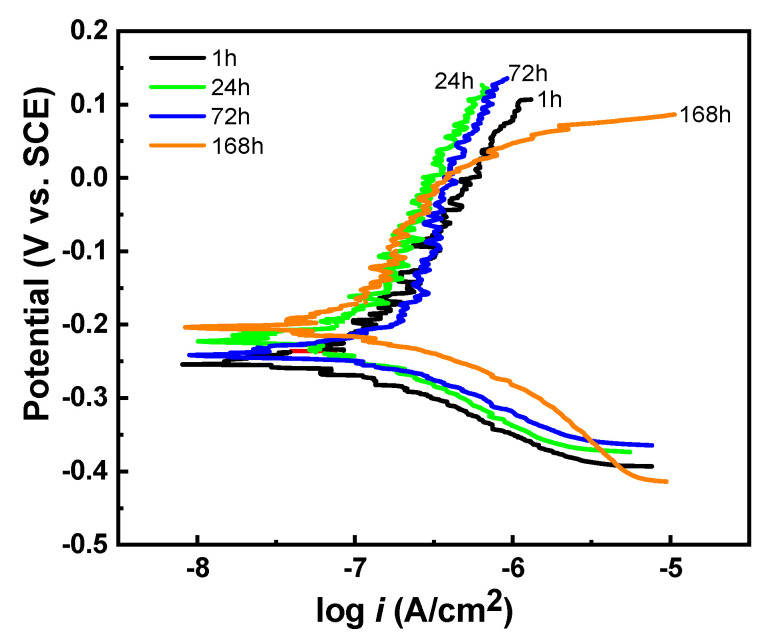
Potentiodynamic polarization curves of 5L films immersed in a 3.5 wt.% NaCl solution for 1, 24, 72, and 168 h.

The stability of the modified zaccagnaite film in a corrosive marine environment was simulated with immersion testing. A 5L film was immersed at increasing durations up to 168 h in a 3.5 wt.% NaCl solution. Figure 7 shows the polarization curves of the film at four different immersion times. Table 3 lists the *E_corr_*, *i_corr_*, and *R_p_* as the average of 3 measurements for the 5L film at various times. The *E_corr_* remained relatively unchanged at approximately −0.240 V vs. SCE from 1 to 72 h. At 168 h the *E_corr_* shifted positively to −0.178 V vs. SCE and the corrosion resistance potential, R*_p_*, began to decrease indicating beginning of corrosion damage at the longer emersion time. The *i_corr_* was also stable starting at 0.12 μA/cm^2^ for 1 h and ending at 0.18 μA/cm^2^ at 168 h. Only the film immersed for 168 h exhibits a pitting potential at the end of the anodic region beginning around 0.0 V suggesting that the film was weakened by the longer exposure to the chloride environment. Current density oscillation is visible again beginning around the corrosion potential and throughout the anodic region.

**Figure 8:**
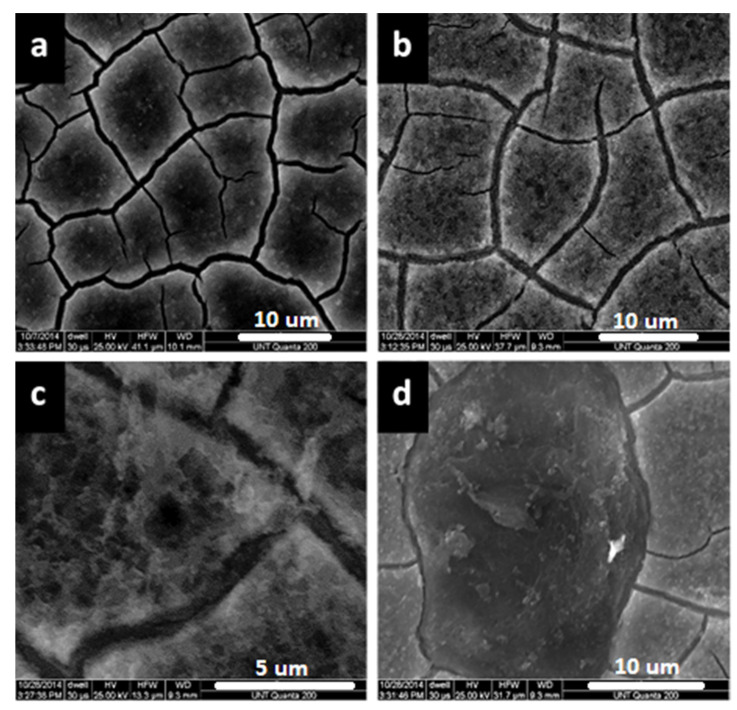
SEM images of 5L film (**a**) before and (**b**) after immersion in 3.5 wt.% NaCl solution for 168 h as well as (**c**) an enlarged image of a defect in the immersed film from (**b**), and (**d**) top section of 5L film removed after 168 h.

SEM images were taken of the 5L film before (Figure 8a) and after immersion in 3.5 wt.% NaCl solution up to 168 h. Figure 8b shows that the aluminum dominated layer on the surface of the film forms pits after immersion of 168 h. These defects range in size from submicron to a couple of microns. These flaws may be evidence of dissociation of the film into hydroxide ions. These defects only affect the top phase and do not appear to penetrate to the substrate because of the stability observed during immersion testing. The top phase may behave as a sacrificial barrier for the underlying coating and possibly releases hydroxide ions. Figure 8d shows a section of the 5L film with the top phase detached after immersion in 3.5 wt.% NaCl solution up to 168 h. While the exposed bottom phase is not free of blemishes, it does not exhibit the pitting that is observed on the top phase.

**Figure 9:**
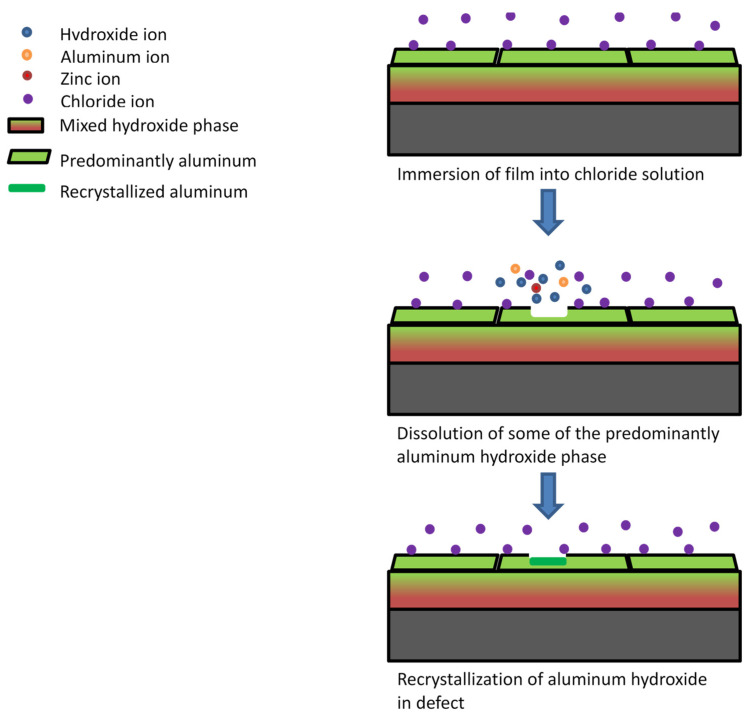
Postulated corrosion mechanism for immersed electrodeposited zaccagnaite film.

The anticorrosion performance of LDH films has been attributed to a multitude of factors. One factor is the high anion exchange capacity of LDH. The nitrate ions in the film have a lower affinity for intercalation than chloride ions. Surface anion exchange with chloride ions traps them and prevents their migration to the substrate. This anion exchange phenomenon has been observed in previous studies [20,21,35]. Another explanation is that the deterioration of the film may release hydroxide ions into the local environment increasing the pH [21]. The release of hydroxide ions can slow down the occurrence of pitting corrosion by reducing the rate of chloride migration to the pit and neutralizing the local solution environment. Previous research has also shown that layered double hydroxides can undergo dissolution/recrystallization or self-healing reactions during the corrosion process [56]. Hydrotalcite has been shown to form a protective amorphous aluminum hydroxide layer to prevent dissolution in mildly acidic solutions [57]. The mostly aluminum hydroxide top phase behaves as a protective coating for the mixed hydroxide phase preventing its dissolution. Figure 9 depicts a proposed corrosion mechanism for the coating after immersion of the film in corrosive media, depicting the sacrificial protection of the aluminum dominate phase and possible crystallization of aluminum hydroxide in pits on the surface. Other explanations for the corrosion resistance of the film is its barrier property. The film is dense and thick enough to prevent the penetration of chloride ions to the substrate surface [21,23]. This can be seen as successive layers are deposited onto the substrate. The corrosion resistance increases from 1 to 5 layers. As the thickness increases (~0.4 to 3 μm), the chloride ions are blocked against attack. Additionally, the film is insulating, resulting in a decrease in the rate of any electrochemical reactions including those involving corrosion. Further study can help determine a comprehensive mechanism of corrosion resistance for the electrodeposited modified zaccagnaite films.

## 4. Conclusions

In this study a facile method was developed to electrochemically deposit modified zaccagnaite films onto a stainless-steel substrate. The electrodeposition occurred in multiple layers in order to minimize defects generated during deposition and drying of the film. XRD and FTIR studies showed the presence of an LDH phase; however, the elemental ratios of Zn:Al were outside typical limits. The metal substrate coated with modified zaccagnaite exhibited higher corrosion resistance than the bare substrate in 3.5 wt.% NaCl solution. Corrosion protection increased with the number of layers deposited. Aluminum concentration in the coating also increased with the number of layers. For the 5-layer coating, *E_corr_* shifted positively ~100 mV and the corrosion current density was reduced by 82% when compared to the bare substrate in 3.5 wt.% NaCl. The 5-layered film also maintained its corrosion resistance while immersed for 168 h in 3.5 wt.% NaCl demonstrating that electrochemically generated modified zaccagnaite is an effective material to reduce corrosion on the substrate surface.

## Figures and Tables

**Table 1:** Elemental composition measured by EDS and thickness of the electrodeposited films (n = 3).

Number of Layers	Zinc Atomic %	Aluminum Atomic %	Thickness (nm)
1	41 ± 4	59 ± 4	431 ± 50
2	24 ± 2	76 ± 2	612 ± 46
5	14 ± 3	86 ± 3	2814 ± 45

**Table 2:** Corrosion potential (*E_corr_*), corrosion current densities (*i_corr_*), and LPR polarization resistance (*R_p_*) measured for all samples in a 3.5 wt.% NaCl solution (n = 3).

Sample	*E_corr_* (V vs. SCE)	*i_corr_* (μA/cm^2^)	*R_p_* (kΩ∙cm^2^)
substrate	−0.363 ± 0.027	0.66 ± 0.01	19 ± 6
1L	−0.269 ± 0.020	0.61 ± 0.19	57 ± 27
2L	−0.240 ± 0.008	0.38 ± 0.11	137 ± 40
5L	−0.237 ± 0.013	0.12 ± 0.01	230 ± 54

**Table 3:** Corrosion potential (*E_corr_*), corrosion current densities (*i_corr_*), and LPR polarization resistance (*R_p_*) measured for a 5L film in 3.5 wt.% NaCl at various immersion times (n = 3).

Immersion Time (h)	*E_corr_* (V vs. SCE)	*i_corr_* (μA/cm^2^)	*R_p_* (kΩ∙cm^2^)
1	−0.239 ± 0.017	0.12 ± 0.02	218 ± 59
24	−0.245 ± 0.031	0.08 ± 0.02	232 ± 37
72	−0.235 ± 0.020	0.18 ± 0.03	309 ± 138
168	−0.178 ± 0.013	0.18 ± 0.02	233 ± 72

## Data Availability

The data presented in this study are available on reasonable request from the corresponding author.
